# Predictive value of initial triage vital signs for in-hospital mortality in acute aortic dissection patients: a retrospective study in the emergency department

**DOI:** 10.3389/fmed.2026.1721657

**Published:** 2026-06-02

**Authors:** Nan Xie, Yixiao Shi

**Affiliations:** 1 Emergency Department of West China Hospital, West China School of Nursing, Sichuan University, Chengdu, China; 2 Disaster Medicine Center, Sichuan University, Chengdu, China; 3 Operating Room of Anesthesia Surgery Center, West China Hospital/West China School of Nursing, Sichuan University, Chengdu, China

**Keywords:** acute aortic dissection, emergency department, in-hospital mortality, triage, vital signs

## Abstract

**Background:**

Acute aortic dissection (AAD) is a life-threatening emergency requiring rapid diagnosis and treatment. Vital signs obtained at triage are immediately available and may provide useful information for early risk stratification.

**Objectives:**

To evaluate whether triage vital signs are independently associated with in-hospital mortality in patients with AAD, with a specific focus on analyzing heart rate as a categorical variable and blood pressure in clinically relevant units.

**Methods:**

We performed a retrospective cohort study of 702 consecutive patients with AAD admitted to the emergency department of a tertiary hospital in China between January 1 and November 23, 2024. Heart rate was categorized as bradycardia (<60 bpm), normal (60–100 bpm), or tachycardia (>100 bpm). Blood pressure was analyzed per 10-mmHg change. Variables significant in univariate analysis (*P* < 0.10) were entered into a multivariate model. Model performance was assessed using the area under the receiver operating characteristic curve (AUC) with 10-fold cross-validation.

**Results:**

Among 702 patients, 293 (41.7%) died during hospitalization. Multivariate logistic regression identified older age (adjusted OR 1.029 per year, 95% CI 1.017–1.041), male sex (adjusted OR 1.495, 95% CI 1.036–2.157), bradycardia (adjusted OR 2.500, 95% CI 1.300–4.800), tachycardia (adjusted OR 1.800, 95% CI 1.100–2.900), lower body temperature (adjusted OR 1.499 per 1 °C decrease, 95% CI 1.082–2.075), and higher diastolic blood pressure (adjusted OR 1.105 per 10-mmHg increase, 95% CI 1.001–1.220) as independent risk factors for in-hospital mortality. The model demonstrated acceptable discrimination (mean cross-validated AUC 0.71, 95% CI 0.68–0.74).

**Conclusion:**

Initial triage vital signs, particularly deviations in heart rate (both low and high), lower body temperature, and higher diastolic blood pressure, are independent predictors of in-hospital mortality in patients with AAD. Assessing these readily available parameters can support early risk stratification in the emergency department.

## 1 Introduction

The emergency department (ED) plays a crucial role in the management of critically ill patients (1). Given the overwhelming number of patients presenting to the ED daily, it is often not feasible for emergency physicians to promptly evaluate all critically ill individuals (2–4). Triage is the first step when patients are admitted to the emergency department (5). Nurses perform triage based on patients’ chief complaints and vital signs and guide them to the appropriate treatment areas (6–8). Due to the intricate nature of complex medical conditions and the abrupt emergence of emergencies in patients, it poses a challenge for triage nurses to recognize the severity of patients first and provide timely interventions (9).

Acute aortic dissection (AAD) is a life-threatening cardiovascular emergency characterized by a high early mortality rate (10, 11). Reports suggest mortality can reach 33% within 24 h and over 50% within 48 h without timely diagnosis and intervention (12). Rapid identification and management of high-risk patients with AAD are essential to improving outcomes.

Despite the extensive body of research reporting factors influencing in-hospital mortality in cases of acute aortic dissection, for example, previous studies have reported that WBC (13, 14), D-dimer (13, 15), CRP (16, 17), LDH (18), serum albumin (19) and intricate computed tomography (CTA) (20) are used for both the identification of acute aortic dissection and the evaluation of its pathological severity. In recent years, various scoring systems have been used to assess the risk of patients in the emergency department, such as NEWS and MEWS (21). However, the application of these diverse scoring systems to predict the prognosis of acute aortic dissection patients is subject to certain limitations. For instance, factors such as excessively small sample sizes and the complexity of employed methodologies contribute to these constraints.

This study aimed to investigate whether triage vital signs can independently predict in-hospital mortality in patients with AAD. Unlike previous research reliant on complex biomarkers or imaging, we focused on refining the analysis of immediately available vital signs–specifically by evaluating heart rate as a categorical variable and blood pressure in clinically meaningful units–to develop a more practical and rapidly applicable prognostic tool in the emergency care setting.

## 2 Materials and methods

### 2.1 Patients and data collection

We retrospectively reviewed all patients diagnosed with acute aortic dissection (AAD) who were admitted to the emergency department of a tertiary hospital in China between January 1 and November 23, 2024. Data were retrospectively collected from our hospital’s Hospital Information System (HIS). Demographic data were collected, including age, sex, and days of hospital stay. Data were extracted from the hospital information system (HIS) and included demographic information (age, sex, length of hospital stay) and triage vital signs: body temperature, respiratory rate (RR), peripheral oxygen saturation (SpO2), systolic blood pressure (SBP), diastolic blood pressure (DBP), heart rate (HR), and Glasgow Coma Scale (GCS) score, which were obtained from Mindray patient monitors (model N17). Triage vital signs were defined as the first measurements recorded at initial patient contact upon arrival in the emergency department, prior to any therapeutic intervention. This study was approved by the hospital’s ethics committee. As the study used retrospective data, informed consent was waived in accordance with institutional policies. All methods were conducted in compliance with relevant guidelines and regulations. Missing data for vital signs were minimal (<2%). Cases with missing key variables were excluded from multivariable analyses, resulting in a final analytic sample of 702 patients.

Standardized Early Warning Score (SEWS): SEWS is a physiologic scoring tool widely used in Chinese emergency departments. It consists of five parameters: respiratory rate, heart rate, systolic blood pressure, level of consciousness, and temperature. Each parameter is assigned 0–3 points based on deviation from normal ranges, and the total score reflects the severity of physiological derangement.

Modified Early Warning Score (MEWS): MEWS includes respiratory rate, heart rate, systolic blood pressure, body temperature, and level of consciousness (AVPU scale). Scores range from 0 to 14, with higher scores indicating greater clinical concern.

National Early Warning Score (NEWS): NEWS comprises seven parameters: respiratory rate, oxygen saturation, need for supplemental oxygen, temperature, systolic blood pressure, heart rate, and level of consciousness. Each variable contributes 0–3 points, with the total score indicating acute illness severity.

The diagnosis of acute aortic dissection was confirmed by computed tomography angiography (CTA) in all included patients.

### 2.2 Measurements

The primary outcome was in-hospital mortality, defined as death occurring in the emergency department, intensive care unit (ICU), or inpatient ward during the same hospital admission. Mortality data were obtained from the hospital information system. This definition was applied consistently across all included patients.

### 2.3 Statistical analysis

Statistical analyses were performed using IBM SPSS Statistics for Windows, Version 22.0 (IBM Corp., Armonk, NY, USA). Continuous variables were expressed as mean ± standard deviation (SD) or median (interquartile range, IQR), and categorical variables as counts and percentages (n, %). Group comparisons between survivors and non-survivors were performed using the Student’s *t*-test or the Mann–Whitney U test for continuous variables, and Pearson’s chi-square test or Fisher’s exact test for categorical variables, as appropriate. Univariate logistic regression was used to screen potential predictors of in-hospital mortality. To enhance clinical interpretability and explore non-linear relationships, specific transformations were applied to key vital signs prior to regression analysis: heart rate was categorized as bradycardia [<60 beats per minute (bpm)], normal heart rate (60–100 bpm), and tachycardia (>100 bpm); and systolic and diastolic blood pressure were analyzed per 10-mmHg change. Variables with *P* < 0.10 in univariate analysis or considered clinically relevant were entered into multivariate logistic regression models to identify independent predictors. To avoid collinearity, composite scores (MEWS and NEWS) were analyzed in separate models. The final multivariable model was constructed using a backward stepwise selection procedure. The discriminative ability of the final model was evaluated using Receiver Operating Characteristic (ROC) curve analysis. To evaluate internal validity, we performed 10-fold cross-validation by randomly partitioning the dataset into 10 subsets. Each subset was used once as a validation set, and the remaining nine subsets as the training set. Model discrimination was assessed by the mean area under the ROC curve (AUC) across all folds. Results from logistic regression are presented as odds ratios (ORs) with 95% confidence intervals (CIs). A two-sided *P* < 0.05 was considered statistically significant. Potential collinearity among variables was assessed during model development, and no evidence of instability in regression estimates was observed.

The analysis was designed to evaluate the prognostic value of pre-treatment triage variables, rather than to construct a comprehensive clinical prediction model incorporating post-admission interventions.

## 3 Results

A total of 702 patients were included in the study, of whom 293 (41.7%) died during hospitalization. Baseline demographic and clinical characteristics are summarized in Table 1. Compared with survivors, patients in the in-hospital death group were older (median [IQR], 61 [53.0–71.0] vs. 54 [46.0–64.0] years, *P* < 0.001) and had a lower proportion of males (70.3% vs. 80.0%, *P* = 0.003). No significant differences were observed between groups in triage level (*P* = 0.057), respiratory rate, or SpO2. Statistically significant but small differences were observed for temperature (36.3 [36.0–36.7] vs. 36.4 [36.1–36.8] °C, *P* = 0.006), heart rate (82 [70–96] vs. 86 [76–96] bpm, *P* = 0.004), diastolic blood pressure (79 [66–92] vs. 83 [71–96] mmHg, *P* = 0.008), and MEWS (1 [1–2] vs. 1 [1–2], *P* = 0.038), though these differences were small and of uncertain clinical significance. GCS score, pain score, SEWS, NEWS, and emergency department length of stay were comparable between groups.

**TABLE 1:** Demographics characteristics of patients diagnosed with acute aortic dissection.

Parameter	Death (*N* = 293)	Survival (*N* = 409)	*P*-value
Age (years), median [IQR]	61 [53.0–71.0]	54 [46.0–64.0]	<0.001
Males, *n* (%)	206 (70.3)	329 (80.0)	0.003
Triage level, *n* (%)			0.057
Red	15 (5.1)	10 (2.4)	
Orange	278 (94.9)	401 (97.6)	
Vital signs			
Temperature (°C), median [IQR]	36.3 [36.0–36.7]	36.4 [36.1–36.8]	0.006
Heart rate (bpm), median [IQR]	82 [70–96]	86 [76–96]	0.004
SBP (mmHg), median [IQR]	137 [114–156]	140 [121–156]	0.052
DBP (mmHg), median [IQR]	79 [66–92]	83 [71–96]	0.008
RR (/min), median [IQR]	20 [20–21]	20 [20–21]	0.705
SPO_2_ (%), median [IQR]	96 [95–98]	96 [95–98]	0.732
GCS score, median [IQR]	14 [13–15]	15 [14–15]	0.220
Pain score, median [IQR]	1 [0–3]	1 [0–3]	0.315
SEWS, median [IQR]	0 [0–1]	0 [0–1]	0.304
MEWS, median [IQR]	1 [1–2]	1 [1–2]	0.038
NEWS, median [IQR]	1 [0–3]	1 [0–3]	0.058
Length of emergency stay (hours), median [IQR]	5.0 [3.0–9.0]	5.0 [3.0–8.0]	0.174

Continuous variables, including age, vital signs, scores, and emergency department length of stay, are presented as median [IQR] and compared using the Mann-Whitney U test. Categorical variables are presented as *n* (%) and compared using the Chi-square test; Fisher’s exact test was used when expected counts were small (e.g., red triage group).

**FIGURE 1:**
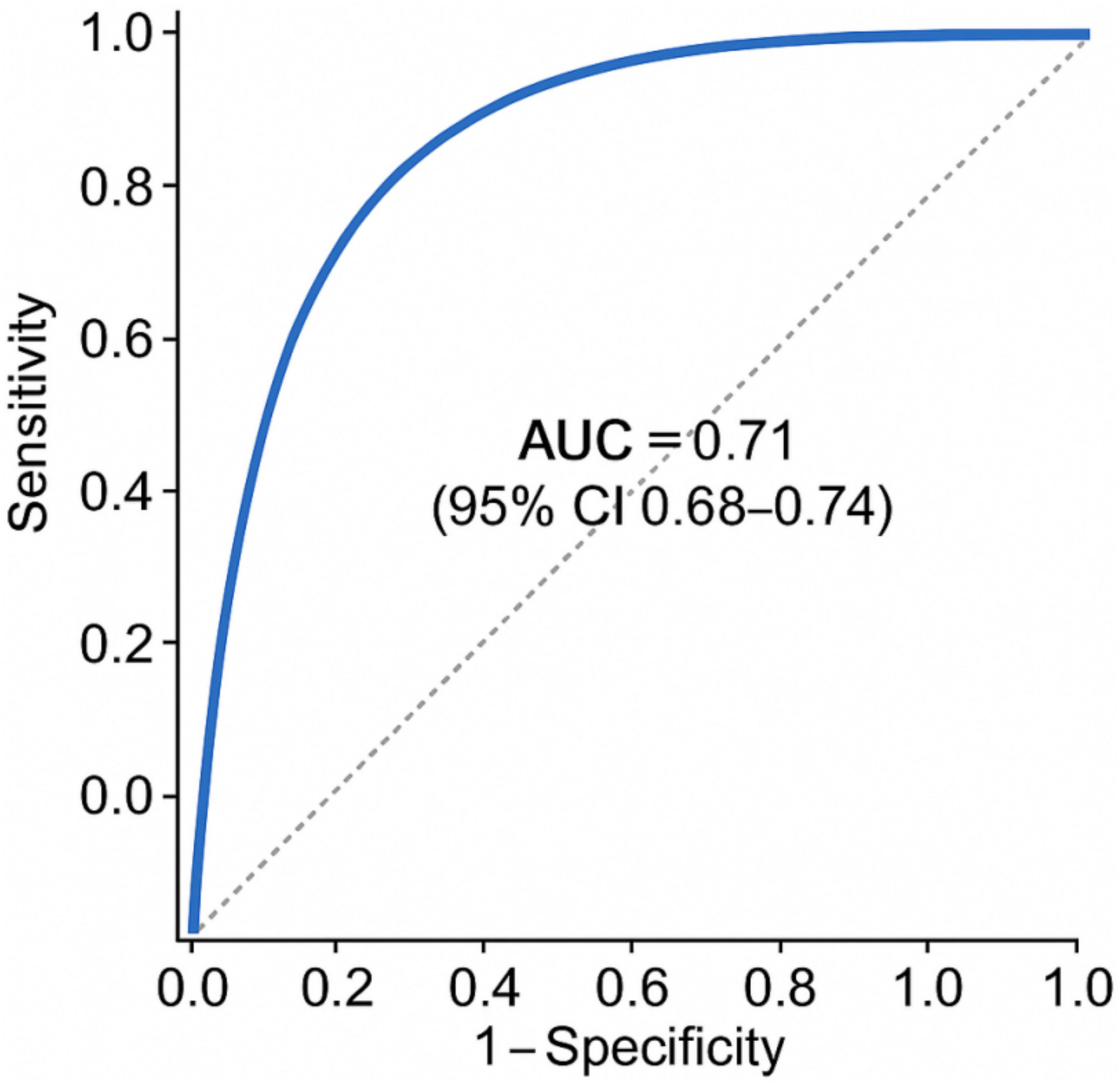
Receiver operating characteristic (ROC) curve of the multivariable logistic regression model for predicting in-hospital mortality.

The discriminative performance of the multivariable model is shown in Figure 1. The model yielded an AUC of 0.71 (95% CI 0.68–0.74) based on 10-fold cross-validation, demonstrating acceptable discrimination for in-hospital mortality prediction among patients with acute aortic dissection.

Univariate logistic regression analysis (Table 2) showed that older age (OR 1.034, 95% CI 1.022–1.034, *P* < 0.001) and male sex (OR 1.684, 95% CI 1.189–2.386, *P* = 0.003) were associated with increased in-hospital mortality. When analyzed as categorical variables, both bradycardia (HR < 60 bpm; OR 2.200, 95% CI 1.400–3.460, *P* < 0.001) and tachycardia (HR > 100 bpm; OR 1.650, 95% CI 1.180–2.310, *P* = 0.003) were significant risk factors compared to normal heart rate. Among other vital signs, lower body temperature (OR 1.541 per 1 °C decrease, 95% CI 1.128–2.105, *P* = 0.006), lower systolic blood pressure (OR 1.063 per 10 mmHg decrease, 95% CI 1.010–1.117, *P* = 0.023), and higher diastolic blood pressure (OR 1.105 per 10 mmHg increase, 95% CI 1.029–1.178, *P* = 0.008) were significantly associated with increased mortality. Triage level, respiratory rate, SpO2, GCS score, pain score, SEWS, and length of emergency stay were not significantly associated with mortality. MEWS (OR 1.229, 95% CI 1.042–1.449, *P* = 0.014) and NEWS (OR 1.104, 95% CI 1.021–1.194, *P* = 0.013) were also significant in univariate analysis.

**TABLE 2:** Univariate logistic regression analyses of relationship between clinical features and in-hospital mortality in patients with acute aortic dissection.

Parameter	OR	95% CI	*P*-value
Age (years)	1.034	1.022–1.034	<0.001
Gender (female vs. male)	1.684	1.189–2.386	0.003
Triage level (Red vs. Orange)	2.153	0.953–4.862	0.065
Vital signs			
Temperature (°C)	1.541	1.128–2.105	0.006
HR (beats/min)			<0.001
Bradycardia (<60)	2.200	1.400–3.460	<0.001
Normal (60–100)	(Reference)	–	–
Tachycardia (>100)	1.650	1.180–2.310	0.003
SBP (mmHg)	1.063	1.010–1.117	0.023
DBP (mmHg)	1.105	1.029–1.178	0.008
RR (/min)	1.011	0.950–1.075	0.735
SPO_2_ (%)	0.996	0.959–1.035	0.849
GCS score	1.073	0.981–1.175	0.125
Pain score	1.001	0.920–1.088	0.991
SEWS	1.109	0.957–1.286	0.170
MEWS	1.229	1.042–1.449	0.014
NEWS	1.104	1.021–1.194	0.013
Length of emergency stay	1.000	0.991–1.009	0.992

OR, odds ratio; CI, confidence interval; Heart rate was categorized as bradycardia [<60 beats per minute (bpm)], normal (60–100 bpm), and tachycardia (>100 bpm). The normal heart rate group served as the reference; Blood pressure [Systolic (SBP) and Diastolic (DBP)] were analyzed per 10-mmHg change. Temperature was analyzed per 1 °C decrease. The OR for continuous variables represents the change in odds per unit/decrease specified.

Multivariate logistic regression (Table 3) identified older age (adjusted OR 1.029 per year, 95% CI 1.017–1.041, *P* < 0.001) and male sex (adjusted OR 1.495, 95% CI 1.036–2.157, *P* = 0.003) as independent predictors of in-hospital mortality. The U-shaped relationship between heart rate and mortality persisted after multivariable adjustment, with both bradycardia (adjusted OR 2.500, 95% CI 1.300–4.800, *P* = 0.006) and tachycardia (adjusted OR 1.800, 95% CI 1.100–2.900, *P* = 0.018) remaining independent risk factors. Among vital signs, lower body temperature (adjusted OR 1.499 per 1 °C decrease, 95% CI 1.082–2.075, *P* = 0.003) and higher diastolic blood pressure (adjusted OR 1.105 per 10 mmHg increase, 95% CI 1.001–1.220, *P* = 0.047) were independent risk factors. Lower systolic blood pressure showed a consistent trend toward increased risk but was not statistically significant in the multivariable model (adjusted OR 1.031 per 10 mmHg decrease, 95% CI 0.956–1.111, *P* = 0.430). NEWS and other variables, including respiratory rate, SpO2, GCS score, pain score, SEWS, triage level, and length of emergency stay, were not significantly associated with mortality.

**TABLE 3:** Multivariate logistic regression analyses to measure in-hospital mortality in patients with acute aortic dissection.

Parameter	Adjusted OR	95% CI	*P*-value
Age (years)	1.029	1.017–1.041	<0.001
Gender (female vs. male)	1.495	1.036–2.157	0.003
Temperature (°C)	1.499	1.082–2.075	0.003
HR (beats/min)			0.001
Bradycardia (<60)	2.500	1.300–4.800	0.006
Normal (60–100)	(Reference)	–	–
Tachycardia (>100)	1.800	1.100–2.900	0.018
SBP (mmHg)	1.031	0.956–1.111	0.430
DBP (mmHg)	1.105	1.001–1.220	0.047
NEWS	1.079	0.989–1.177	0.086

OR, odds ratio; CI, confidence interval; Heart rate was categorized as bradycardia [<60 beats per minute (bpm)], normal (60–100 bpm), and tachycardia (>100 bpm). The normal heart rate group served as the reference; Blood pressure [Systolic (SBP) and Diastolic (DBP)] were analyzed per 10-mmHg change. Temperature was analyzed per 1 °C decrease. The OR for continuous variables represents the change in odds per unit/decrease specified.

**FIGURE 2:**
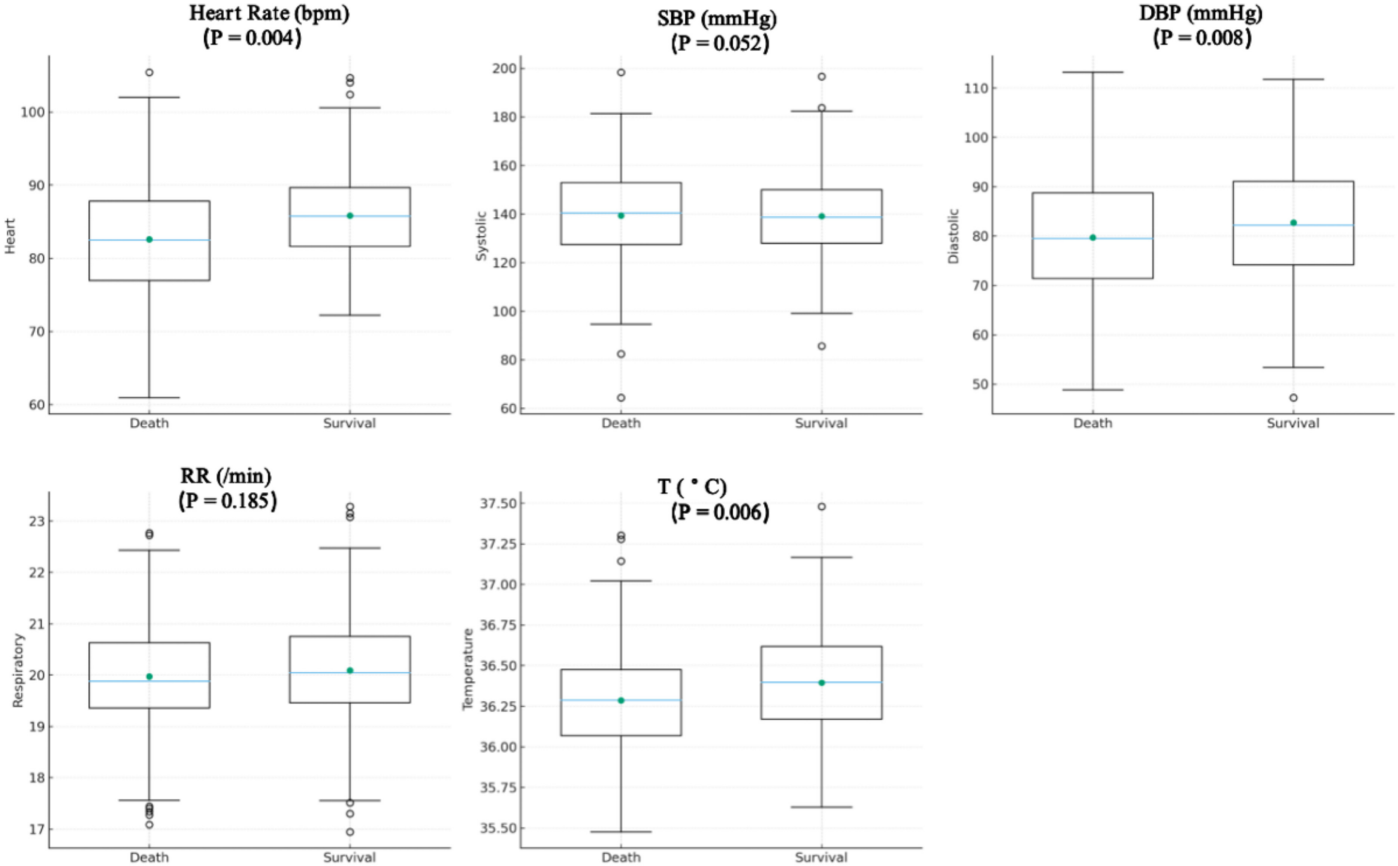
Boxplots of vital signs stratified by in-hospital outcome. Boxplots comparing the distribution of HR, SBP, DBP, RR and temperature, between survivors and non-survivors. The thick horizontal line indicates the median, the box represents the interquartile range, and whiskers show the range. Circles denote outliers. *P*-values were calculated using the Mann–Whitney U test.

The distributional differences of key triage vital signs are illustrated in Figure 2. Compared with survivors, non-survivors had significantly lower HR (median [IQR]: 82 [26] vs. 86 [20] beats/min, *P* = 0.004), DBP (79 [26.5] vs. 83 [24.8] mmHg, *P* = 0.008), and temperature (36.3 [0.7] vs. 36.4 [0.7] °C, *P* = 0.006). SBP showed a trend toward lower values in non-survivors but did not reach statistical significance (136.5 [42.3] vs. 140.0 [35.0] mmHg, *P* = 0.052). No significant difference was found in RR between the two groups (20 [3] vs. 20 [2] breaths/min, *P* = 0.185). These findings highlight subtle yet significant differences in vital signs between groups, underscoring their potential value in risk stratification.

## 4 Discussion

The emergency department plays a pivotal role in managing critically ill patients, given its responsibility for the judicious allocation of resources through effective triage (22). This study aimed to investigate the potential of initial vital signs as a tool for assessing the severity of acute aortic dissection in patients and as an early indicator of mortality risk prior to definitive diagnostic confirmation. Our findings shed light on the importance of specific vital signs in prognosticating the outcomes of acute aortic dissection cases.

Our results confirmed several established demographic risk factors. Consistent with existing literature, older age and male sex were independent predictors of in-hospital mortality (23–26), highlighting patient subgroups that warrant heightened vigilance upon ED arrival.

The most significant finding regarding vital signs pertains to heart rate The observed U-shaped association may reflect complex physiological responses in acute aortic dissection; however, underlying factors such as medication use or cardiac tamponade could not be evaluated. Specifically, both bradycardia (HR < 60 bpm) and tachycardia (HR > 100 bpm) were independent risk factors compared to a normal heart rate. This underscores the unique pathophysiology of AAD. Bradycardia may signal hemodynamic decompensation, cardiac tamponade, or excessive vagal tone, while tachycardia could reflect severe pain, sympathetic overdrive, or hypovolemia. Both extremes represent a state of profound circulatory instability that portends a worse outcome, indicating that a lower heart rate in this population does not necessarily indicate stability but may, in fact, be an ominous sign.

Regarding other vital signs, our study quantified their independent prognostic value using clinically relevant units. We found that lower body temperature (per 1 °C decrease) was a significant independent risk factor. While the precise mechanism requires further investigation, hypothermia may be a marker of neurohormonal dysregulation, systemic inflammatory response, or peripheral circulatory failure in the most critically ill patients. Furthermore, our analysis of blood pressure yielded distinct insights for systolic and diastolic components. Lower systolic blood pressure (per 10 mmHg decrease) showed a consistent trend toward increased risk, aligning with the conventional understanding of hypotension as a marker of shock or tamponade. More intriguingly, higher diastolic blood pressure (per 10 mmHg increase) was identified as an independent risk factor. This finding appears counterintuitive and should be interpreted with caution. Rather than indicating a direct causal relationship, it may reflect the complex hemodynamic state in patients with AAD. Further studies are needed to validate this observation.

Our reanalysis demonstrated that both bradycardia and tachycardia were associated with increased mortality, indicating a U-shaped rather than linear association between HR and outcome. This finding aligns with the physiological understanding that both extremes of heart rate reflect hemodynamic instability in AAD. Although the quadratic term was not statistically significant, this trend deserves further validation in larger cohorts.

Comparing our results with previous studies, we observed both similarities and differences in predictors of in-hospital mortality. While previous research has reported associations between inflammatory markers and AAD outcomes (27, 28), our study demonstrates that simple, immediate triage vital signs provide substantial prognostic information. Additionally, the incorporation of scoring systems such as NEWS and MEWS in emergency departments has shown promise in risk assessment (29, 30). However, in our cohort, the NEWS score was not an independent predictor of mortality after adjusting for specific vital signs and demographics. Interestingly, factors commonly used in other scoring systems, such as NEWS and MEWS, including respiratory rate and oxygen saturation, did not show significant associations with in-hospital mortality in our study (31, 32). This discrepancy, coupled with our findings on heart rate and diastolic pressure, reinforces the notion that the pathophysiology of AAD is distinct, with specific cardiovascular parameters play a more pivotal role in determining patient outcomes (33).

Although vital signs are universally and routinely measured, evidence quantifying their independent prognostic value in AAD has been limited. Our study is among the first to delineate these relationships clearly using real-world triage data and clinically oriented analyses. These findings suggest that integrating the assessment of these specific vital signs–particularly recognizing extremes of heart rate, low body temperature, and elevated diastolic pressure–into early-warning protocols could help emergency clinicians rapidly identify high-risk AAD patients even before definitive imaging confirmation, enabling expedited surgical consultation or intensive care unit admission.

From a clinical perspective, our findings empower emergency providers to use routinely collected triage vital signs for the immediate risk stratification of AAD patients. The recognition that bradycardia, not just tachycardia, is a critical risk factor, and that elevated diastolic pressure provides unique prognostic information, offers nuanced, actionable insights beyond general early warning scores. From a public health standpoint, this study highlights the potential of a low-cost, universally available tool–vital signs interpreted through an AAD-specific lens–to guide timely care. This is particularly relevant for resource-limited settings and could form the basis for standardized AAD-risk protocols in emergency departments, ultimately aiming to improve equity and outcomes for this time-sensitive condition.

Our study has several limitations that should be acknowledged. First, as a single-center retrospective study, selection and information bias cannot be excluded. Second, unmeasured confounders such as pre-hospital interventions (e.g., treatment timing, surgical strategy, and medication use) and comorbidities were not available. Third, although internal cross-validation was performed, external validation is still required. Fourth, the observational design precludes causal inference. Finally, our analysis assumed a linear relationship between blood pressure and log-odds of mortality. Future studies with larger sample sizes could valuably explore potential non-linear or threshold effects.

## 5 Conclusion

In this cohort of patients with acute aortic dissection, initial triage assessment provides critical prognostic information. We identified older age, male sex, and deviations from normal in key hemodynamic parameters as independent predictors of in-hospital mortality. Specifically, the risk of death was significantly higher in patients with bradycardia or tachycardia, lower body temperature, and higher diastolic blood pressure. A trend toward increased risk was also observed with lower systolic blood pressure. These findings underscore the importance of early identification of high-risk patients based on simple clinical parameters available at presentation. Implementing triage protocols that recognize the prognostic significance of these specific parameters, particularly the U-shaped relationship of heart rate, may assist in early risk stratification before definitive imaging, rather than directly altering established diagnostic or treatment pathways.

## Data Availability

The original contributions presented in this study are included in the article/supplementary material, further inquiries can be directed to the corresponding author.

## References

[1] Mathews K Durst M Vargas-Torres C Olson A Mazumdar M Richardson L. Effect of emergency department and ICU occupancy on admission decisions and outcomes for critically Ill patients. *Crit Care Med.* (2018) 46:720–7. 10.1097/CCM.0000000000002993 29384780 PMC5899025

[2] Angus D. Caring for the critically ill patient: challenges and opportunities. *JAMA.* (2007) 298:456–8. 10.1001/jama.298.4.456 17652301

[3] Santos JLGD Lima MADDS Pestana AL Garlet ER Erdmann AL. Challenges for the management of emergency care from the perspective of nurses. *Acta Paulista de Enfermagem.* (2013) 26:136–43. 10.1590/S0103-21002013000200006

[4] Yarmohammadian MH Rezaei F Haghshenas A Tavakoli N. Overcrowding in emergency departments: a review of strategies to decrease future challenges. *J Res Med Sci.* (2017) 22:23. 10.4103/1735-1995.200277 28413420 PMC5377968

[5] Ebrahimi M Mirhaghi A Mazlom R Heydari A Nassehi A Jafari M. The role descriptions of triage nurse in emergency department: a delphi study. *Scientifica.* (2016) 2016:5269815. 10.1155/2016/5269815 27382500 PMC4921622

[6] Wolf LA Delao AM Perhats C Moon MD Zavotsky KE. Triaging the emergency department, not the patient: United States emergency nurses’ experience of the triage process. *J Emerg Nurs.* (2018) 44:258–66. 10.1016/j.jen.2017.06.010 28750891

[7] Rowe BH Villa-Roel C Guo X Bullard MJ Ospina M Vandermeer B et al. The role of triage nurse ordering on mitigating overcrowding in emergency departments: a systematic review. *Acad Emerg Med.* (2011) 18:1349–57. 10.1111/j.1553-2712.2011.01081.x 21692901

[8] Christ M Grossmann F Winter D Bingisser R Platz E. Modern triage in the emergency department. *Dtsch Arztebl Int.* (2010) 107:892–8. 10.3238/arztebl.2010.0892 21246025 PMC3021905

[9] Moll H. Challenges in the validation of triage systems at emergency departments. *J Clin Epidemiol*. (2010) 63:384–8. 10.1016/j.jclinepi.2009.07.009 19875271

[10] Clouse W Hallett J Schaff H Spittell P Rowland C Ilstrup D et al. Acute aortic dissection: population-based incidence compared with degenerative aortic aneurysm rupture. *Mayo Clin Proc.* (2004) 79:176–80. 10.4065/79.2.176 14959911

[11] Glower DD Speier RH White WD Smith LR Rankin JS Wolfe WG. Management and long-term outcome of aortic dissection. *Ann Surg.* (1991) 214:31–41. 10.1097/00000658-199107000-00006 2064469 PMC1358411

[12] Howard DP Banerjee A Fairhead JF Perkins J Silver LE Rothwell PM. Population-based study of incidence and outcome of acute aortic dissection and premorbid risk factor control: 10-year results from the Oxford vascular study. *Circulation.* (2013) 127:2031–7. 10.1161/CIRCULATIONAHA.112.000483 23599348 PMC6016737

[13] Sbarouni E Georgiadou P Marathias A Geroulanos S Kremastinos DT. D-dimer and BNP levels in acute aortic dissection. *Int J Cardiol.* (2007) 122:170–2. 10.1016/j.ijcard.2006.11.056 17234284

[14] Wen D Wu H-Y Jiang X-J Zhang H-M Zhou X-L Li J-J et al. Role of plasma C-reactive protein and white blood cell count in predicting in-hospital clinical events of acute type A aortic dissection. *Chinese Med J.* (2011) 124:2678–82. 22040423

[15] Xie N Zhang W Li H Zhou J Yang X Zou L et al. Admission values of plasma biomarkers predict the short-term outcomes in acute aortic dissection. *Heart Surg Forum.* (2021):E048–054. 10.1532/hsf.3417 33635247

[16] Vrsalović M Vrsalović Presečki A. Admission C-reactive protein and outcomes in acute aortic dissection: a systematic review. *Croatian Med J.* (2019) 60:309–15. 10.3325/cmj.2019.60.309 PMC6734568 31483116

[17] Wen D Du X Dong J-Z Zhou X-L Ma C-S. Value of D-dimer and C reactive protein in predicting inhospital death in acute aortic dissection. *Heart.* (2013) 99:1192–7. 10.1136/heartjnl-2013-304158 23813850

[18] He H Chai X Zhou Y Pan X Yang G. Association of lactate dehydrogenase with in-hospital mortality in patients with acute aortic dissection: a retrospective observational study. *Int J Hypertension.* (2020) 2020:1347165. 10.1155/2020/1347165 PMC6969996 31969993

[19] Gao Y Li D Cao Y Zhu X Zeng Z Tang L. Prognostic value of serum albumin for patients with acute aortic dissection: a retrospective cohort study. *Medicine.* (2019) 98:e14486. 10.1097/MD.0000000000014486 30732220 PMC6380797

[20] Moon M Greenberg R Morales J Martin Z Lu Q Dowdall J et al. Computed tomography-based anatomic characterization of proximal aortic dissection with consideration for endovascular candidacy. *J Vasc Surg.* (2011) 53:942–9. 10.1016/j.jvs.2010.10.067 21345636

[21] Azijli K Minderhoud T Mohammadi P Dekker R Brown V Attaye T et al. A prospective, observational study of the performance of MEWS, NEWS, SIRS and qSOFA for early risk stratification for adverse outcomes in patients with suspected infections at the emergency department. *Acute Med.* (2021) 20:116–24. 10.52964/amja.0851 34190738

[22] Bahari A Asadi F. A simulation optimization approach for resource allocation in an emergency department healthcare unit. *Glob Heart.* (2020) 15:14. 10.5334/gh.528 32489787 PMC7218783

[23] Meccanici F Gökalp AL Thijssen CG Mokhles MM Bekkers JA van Kimmenade R et al. Male–female differences in acute thoracic aortic dissection: a systematic review and meta-analysis. *Interactive CardioVasc Thoracic Surg.* (2022) 34:616–27. 10.1093/icvts/ivab270 PMC8972321 34664071

[24] Liang N Genovese E Al-Khoury G Hager E Makaroun M Singh M. Effects of gender differences on short-term outcomes in patients with type B aortic dissection. *Ann Vasc Surg*. (2017) 38:78–83. 10.1016/j.avsg.2016.06.006 27521832 PMC5164863

[25] Rylski B Georgieva N Beyersdorf F Büsch C Boening A Haunschild J et al. Gender-related differences in patients with acute aortic dissection type A. *J Thorac Cardiovasc Surg.* (2021) 162:528–535.e1. 10.1016/j.jtcvs.2019.11.039 31926709

[26] Nienaber CA Fattori R Mehta RH Richartz BM Evangelista A Petzsch M et al. Gender-related differences in acute aortic dissection. *Circulation.* (2004) 109:3014–21. 10.1161/01.CIR.0000130644.78677.2C 15197151

[27] Zhang C Fu Z Bai H Lin G Shi R Chen X et al. Admission white blood cell count predicts post-discharge mortality in patients with acute aortic dissection: data from the MIMIC-III database. *BMC Cardiovasc Disord.* (2021) 21:462. 10.1186/s12872-021-02275-0 34563109 PMC8466640

[28] Fan X Huang B Lu H Zhao Z Lu Z Yang Y et al. Impact of admission white blood cell count on short-and long-term mortality in patients with type A acute aortic dissection: an observational study. *Medicine.* (2015) 94:e1761. 10.1097/MD.0000000000001761 26496299 PMC4620771

[29] Covino M Sandroni C Della Polla D De Matteis G Piccioni A De Vita A et al. Predicting ICU admission and death in the emergency department: a comparison of six early warning scores. *Resuscitation.* (2023) 190:109876. 10.1016/j.resuscitation.2023.109876 37331563

[30] Holmström L Zhang F Ouyang D Dey D Slomka P Chugh S. Artificial intelligence in ventricular arrhythmias and sudden death. *Arrhythm Electrophysiol Rev.* (2023) 12:e17. 10.15420/aer.2022.42 37457439 PMC10345967

[31] Williams T Tohira H Finn J Perkins G Ho K. The ability of early warning scores (EWS) to detect critical illness in the prehospital setting: a systematic review. *Resuscitation.* (2016) 102:35–43. 10.1016/j.resuscitation.2016.02.011 26905389

[32] Bellou V Belbasis L Konstantinidis A Tzoulaki I Evangelou E. Prognostic models for outcome prediction in patients with chronic obstructive pulmonary disease: systematic review and critical appraisal. *BMJ.* (2019) 367:l5358. 10.1136/bmj.l5358 31585960 PMC6776831

[33] Milewicz DM Ramirez F. Therapies for thoracic aortic aneurysms and acute aortic dissections: old controversies and new opportunities. *Arteriosclerosis Thrombosis Vascular Biol.* (2019) 39:126–36. 10.1161/ATVBAHA.118.310956 PMC6398943 30651002

